# Insight into the Biological Roles and Mechanisms of Phytochemicals in Different Types of Cancer: Targeting Cancer Therapeutics

**DOI:** 10.3390/nu15071704

**Published:** 2023-03-31

**Authors:** Taghreed A. Majrashi, Saad Ali Alshehri, Abdulrhman Alsayari, Abdullatif Bin Muhsinah, Mohammad Alrouji, Asma M. Alshahrani, Anas Shamsi, Akhtar Atiya

**Affiliations:** 1 Department of Pharmacognosy, College of Pharmacy, King Khalid University (KKU), Guraiger, Abha 62529, Saudi Arabia; 2 Complementary and Alternative Medicine Unit, King Khalid University (KKU), Abha 62529, Saudi Arabia; 3 Department of Medical Laboratories, College of Applied Medical Sciences, Shaqra University, Shaqra 11961, Saudi Arabia; 4 Department of Clinical Pharmacy, College of Pharmacy, King Khalid University (KKU), Abha 62529, Saudi Arabia; 5 Center for Medical and Bio-Allied Health Sciences Research, Ajman University, Ajman P.O. Box 346, United Arab Emirates

**Keywords:** phytochemicals, phenolic compounds, cancer therapeutics, natural products, cell signaling

## Abstract

Cancer is a hard-to-treat disease with a high reoccurrence rate that affects health and lives globally. The condition has a high occurrence rate and is the second leading cause of mortality after cardiovascular disorders. Increased research and more profound knowledge of the mechanisms contributing to the disease’s onset and progression have led to drug discovery and development. Various drugs are on the market against cancer; however, the drugs face challenges of chemoresistance. The other major problem is the side effects of these drugs. Therefore, using complementary and additional medicines from natural sources is the best strategy to overcome these issues. The naturally occurring phytochemicals are a vast source of novel drugs against various ailments. The modes of action by which phytochemicals show their anti-cancer effects can be the induction of apoptosis, the onset of cell cycle arrest, kinase inhibition, and the blocking of carcinogens. This review aims to describe different phytochemicals, their classification, the role of phytochemicals as anti-cancer agents, the mode of action of phytochemicals, and their role in various types of cancer.

## 1. Introduction

**Figure 1:**
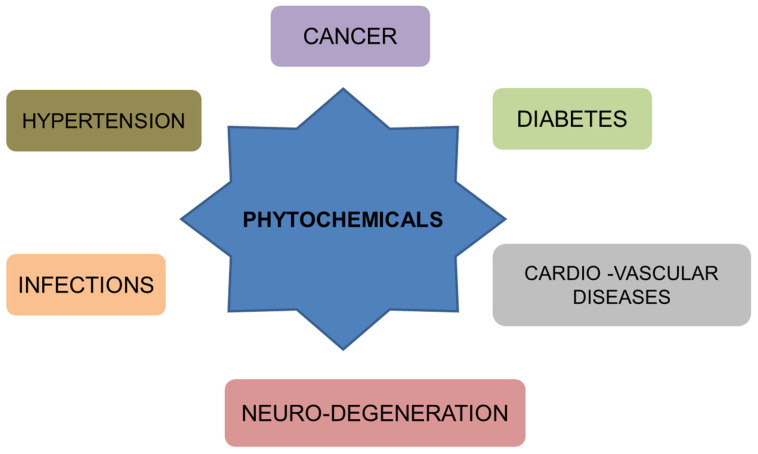
Therapeutic potential of phytochemicals in different human ailments.

Cancer has emerged as a major health issue and is known to be the most prevalent disease after cardiovascular diseases. In 2018, around 18 million cancer cases emerged globally; this number is estimated to increase to more than 23 million new cases annually by 2030 [1]. The disease is hard to treat and has a high chance of reversal after treatment. The presently accessible cancer treatment involves the removal by surgery and radiotherapy of the biomass accumulated by the cancer, and the procedure is followed by chemotherapy. The chemotherapeutic treatments include various antimetabolites, DNA-interacting agents, hormones, and molecular targeting agents [2]. Chemotherapy is effective, yet it faces major challenges such as chemoresistance by cancer cells, recurrence, and toxicity exerted on normal cells, ultimately impairing life quality. Thus, many rely on complementary and alternative medicine (CAM) [3]. The primary area of research in anti-cancer therapy is chemoprevention, focusing on numerous aspects ranging from nutritional to pharmacological factors. To tackle the problems associated with current therapies in cancer treatment, there is still a search for anticancer agents with enhanced efficiency and minimal side effects [4]. A major task in cancer management is overpowering chemoresistance and the failure of current chemotherapies. Resistance to chemotherapy is associated with modulated metabolism in cancer [5]. The compounds have also gained FDA approval for administration in regulated amounts [6]. Figure 1 depicts the use of natural compounds to treat various human ailments. Therefore, the metabolic modulations render the cancer cells more resistant to chemotherapy and increase at an enhanced rate [7]. Resistance to drugs in cancer is closely associated with an increase in glycolysis, even under sufficient oxygen conditions [8]. The colon cancer cells exhibiting chemoresistance show reduced production of ATP and increased aerobic glycolysis. Recent research focuses on identifying the genes responsible for providing chemoresistance and finding a safe and effective method to overcome cancer and drug resistance.

Phytochemicals are naturally occurring chemicals derived from plants, and various naturally occurring compounds have shown promising results in various human ailments with no adverse effects. Phytochemicals and their derivatives are biologically active compounds and have shown anti-cancer effects [9,10,11,12]. The development of phytochemical-based anti-cancer agents involves the extraction, separation, and purification of different compounds. The separated compounds are further tested on various cell lines in vitro and in vivo. The traditional knowledge that involved the selection of plants, collection methods, preparation of drugs, and their use was passed on from generation to generation. The drugs were used in various forms, such as teas, powders, formulations, decoctions, etc. [13,14], until the 18th century. The first breakthrough in drug discovery was the isolation of an analgesic from the plant *Papaver somniferum*, known as morphine. Afterward, many other drugs were derived from plants, including cocaine from *Erythroxylum coca*, aspirin from *Salix sp*, quinine from *Cinchona officinalis*, digitoxin from Digitalis purpurea, and many more having pharmacological activities [13,15,16]. Some of the most widely used anti-cancer agents derived from plant sources are Taxol, Demecolcine, Colcemid, Paclitaxel, etc. [17].

Plants are a rich source of phytochemicals and chemical entities and have many therapeutic applications [18]. Although modern and easy chemotherapeutic drugs offer first-line treatment, the problem associated with them is their various side effects. Therefore, researchers are interested in treatments with minimal side effects [19]. The phytochemicals effectively target different cancers and minimize various hallmarks of cancer, reducing its intensity. The chemo-protective roles of the phytochemicals are exerted by modulating the signaling pathways involved in cancer. This is found to have connections with the apoptosis induction and suppression of the epithelial to mesenchymal EMT, thereby resulting in the blockage of the metastatic behavior of cancer cells [19]. The phytochemicals interfere with various signaling cascades such as MAPK pathway, nuclear factor kappa B (NF-κB) signaling, PI3K-mTOR pathway, etc. [20,21]. The natural compounds also interfere with some of the protein kinases overexpressed in cancers, such as MARKs, AMPKs, PDKs, and SPHKs. Inhibition of these protein kinases with natural compounds provides a safe and effective approach in cancer therapeutics [11,20,22,23].

The phytochemicals also target the cancer stem cells, affecting the cells’ sensitivity toward chemotherapeutic drugs [24]. Phytochemicals have also shown modulatory metabolic properties in cancer cells by governing different steps in the cancer signaling pathway [25]. The chemicals can also modulate the membrane potential of the mitochondrial membrane and control the mitochondrial pathways [26]. The natural compounds have also shown immunoprotective effects. The phytochemicals modulate the immunosuppressive behavior of the cancer cells by modulating the T-regulatory (Treg) cells. Some of the natural products with immune-modulatory effects are [27]. This review elaborates on the classification of phytochemicals and the anti-cancer roles of phytochemicals.

## 2. Phytochemicals

Phytochemicals are very active constituents and are abundant in nature. As mentioned above, they are grouped and have significant roles in preventing various diseases. The use of these phytochemicals is done in a combination of multiple phytochemicals and other drugs as well [9,10,11,12,13,14]. Phytochemicals exhibit a wide range of therapeutic roles, including antioxidant, anti-inflammatory, anti-diabetic, analgesic, anti-cancer, neuroprotective, and anti-microbial activities [24,25,26,27,28,29,30,31,32,33,34,35,36,37,38,39,40,41,42,43,44,45,46,47]. Phytochemicals are an essential source for the development and discovery of new potent drugs [48]. The effects include apoptosis, alterations in signaling pathways, cell cycle blockage, DNA damage, etc. [28].

**Figure 2:**
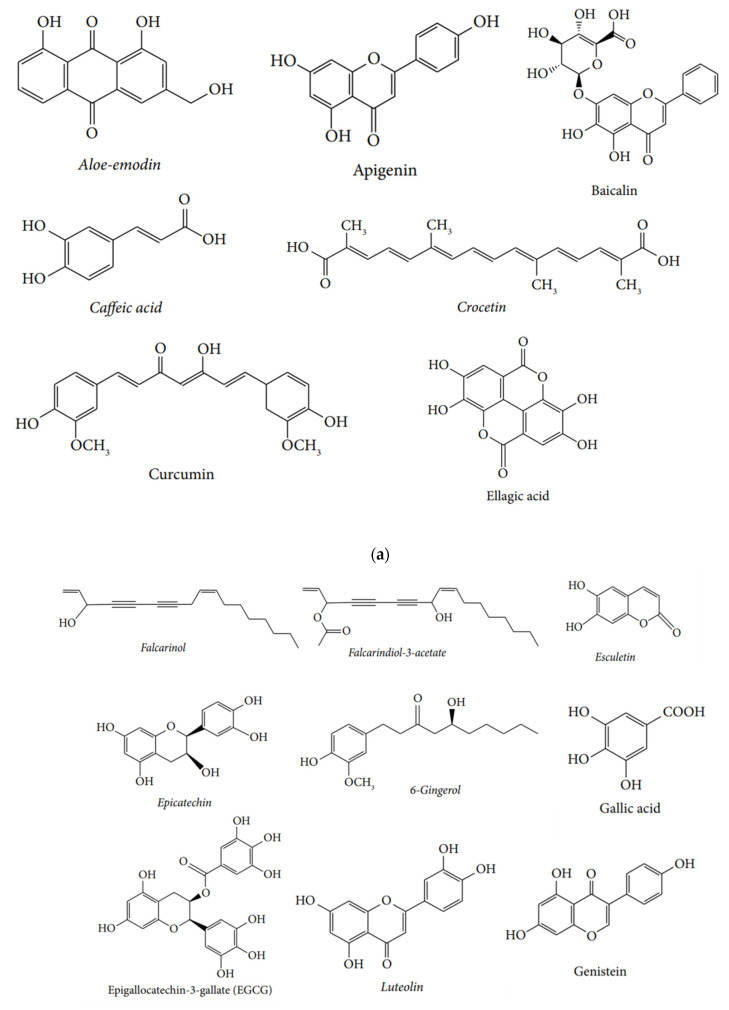

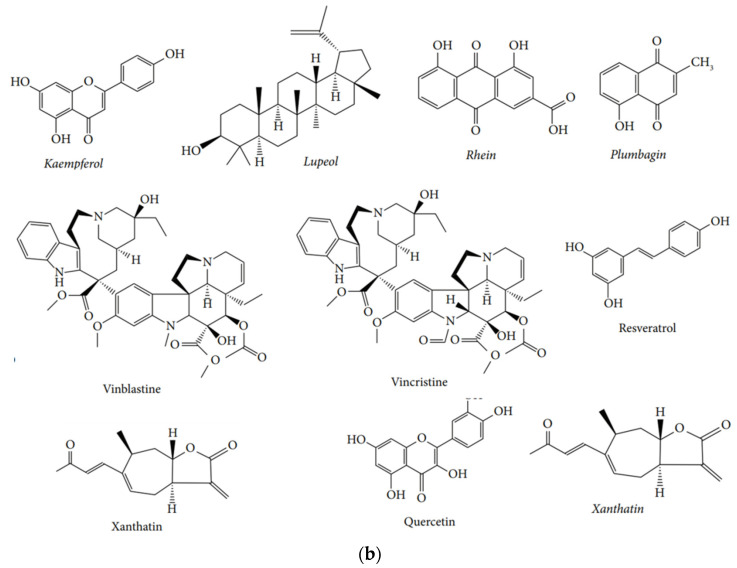
(**a**) Different phytochemicals used as therapeutic agents: aloe-emodlin, apigenin, baicalin, caffeic acid, crocetin, curcumin, and ellagic acid; (**b**) Different phytochemicals used as therapeutic agents: epigallocatechin-3-gallate (EGCG). luteolin, genistein, kaempferol, lupeol, Rhein, plumbagin, vinblastine, vincristeine, resveratrol, xanthatin, quercetin, and xanthin.

Various anti-cancer agents originating from plant sources have found their use and approval, such as vincristine, taxol, paclitaxel, camptothecin derivatives, chinconine, etc. [29]. Various studies have shown that the compound curcumin (originating from the roots of *Curcuma longa* L.) shows anticancer effects by inducing apoptosis, thereby inhibiting the proliferation of cancer cells and resulting in cell cycle arrest in various cancer cell lines [30]. Some organosulfur components obtained from the *Allium sativum* L. plant, such as S-allylcysteine, show retarding effects on the growth of the tumor in various in vivo models [31]. Epigallocatechin-3-gallate (EGCG) from green tea also shows anti-cancer and anti-microbial effects and is a very vital phytochemical [32,33]. The *Catharanthus roseus* (L.) plant is a rich source of alkaloids such as vinblastine and vincristine, which are used in the current treatment of various types of cancer such as breast cancer (BC), lung cancer, lymphomas, and leukemia [34]. Gymnemagenol is obtained from *Gymnema sylvestre* and shows promising anti-cancer potential against hepatic cancer cell lines. MTT assay to estimate the anti-proliferative activity of the phytochemical against HeLa cell lines was performed, and gymnemagenol showed an IC_50_ value of 37 μg/m [35]. In another study, baicalein, isolated from *Oroxylumindicum, exhibited an* antitumor effect on human cancer cell lines by inhibiting the HL-60 cell line proliferation [36]. Antitumor activity of various phytochemicals has been reported and is undergoing clinical trials. The compounds are at different stages of chemical trials for cancer. Table 1 lists the phytochemicals tested in clinical trials. Some of the phytochemicals showing therapeutic effects are shown in Figure 2a,b.

Polyphenols have no exact classification, yet as they have diverse structures and are abundant in nature. The phytochemicals are generally classified as primary and secondary metabolites, as per their roles in plant metabolisms. The class of primary metabolites includes common sugars, nucleic acids, and proteins, all of which play an essential role in the basic survival of the plant. The secondary metabolites are plant chemicals that provide extra advantages over basic survival strategies such as flowering, defense mechanisms, anti-microbial agents, etc. [37,38].

**Figure 3:**
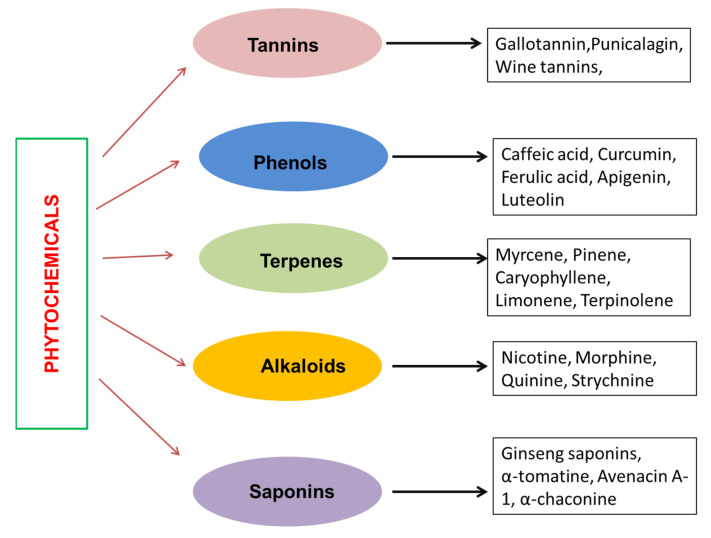
The diverse phytochemicals originating from plant sources are distinguished into five major classes based on their chemical structure and properties. The figure illustrates the different classes and gives examples of each type.

Secondary metabolites mainly consist of lignans, alkaloids, terpenes, phytoalexins, triterpenes, steroids, stilbenoids, bibenzyls, phenols, flavonoids, etc. [39]. Phenolics are known to be the most prevalent and structurally diverse phytochemicals. Figure 3 depicts the classification of phytochemicals.

## 3. Phenolic Compounds and Their Role in Cancer Management

Phenolic compounds are the major components of phytochemicals that are widely distributed in the plant kingdom [56]. They aid in defense mechanisms as secondary metabolites. Additionally, phenolic compounds benefit humans in multiple ways; their antioxidant properties are widely considered a significant benefit for humans in this disease era. Flavonoids, phenolic acids, and polyphenols are three major groups of dietary phenolics. Flavonoids are a large group of phenols that occur ubiquitously as aglycones, glucosides, and methylated derivatives [57]. Many thousands of flavonoids, such as those found in fruits, vegetables, tea, and coffee, have been known to occur abundantly as a part of our diet [58]. Flavonoids have been used successfully in treating ailments since ancient times and have found their uses to date. Flavonoids usually occur in conjugation with sugars and are classified further as mono-, di-, and oligo-glycosides. Flavonoids are gaining attention due to their effect on various biological and pharmacological functions. Some effects exerted on biological functions include cytotoxic effects against cancer cell lines, anti-tumor effects, anti-inflammatory effects, and anti-microbial effects. Apart from therapeutic effects, the group of phytochemicals is known for its potent antioxidant activity, which plays a vital role in protection from the harmful effects of free radicals and reactive oxygen species (ROS). The phenolic acids form diverse groups and are abundantly distributed, such as hydroxylbenzoic acid (HBA) and hydroxycinnamic acid (HCA), and have one carboxylic acid functional group. HCAs are simple esters with an attached glucose or hydroxycarboxylic acid group. The phenolic compounds produced by plants have a different molecular structure, well known by the presence of hydroxylated aromatic rings [59]. The compounds are known for their antioxidant properties that prevent oxidative damage against ROS, thereby playing a vital role in neurodegeneration, cardiovascular diseases (CVDs), cancers, and many more. Tumor cells have a higher generation of ROS than normal cells and, therefore, are targeted by these compounds [60].

The importance of phenolic compounds is attributed to their effectiveness against the proliferation of various human cancer cell lines (HCCL) [61,62]. Cinnamic acid (CA) is a monocarboxylic acid derived from acrylic acid with a phenyl substituent. According to published literature, CA reduced the cell proliferation of the melanoma cell line (HT-44) with an IC_50_ value of 2.4 mM and inhibited the growth of the HT-44 cells by inhibiting the cells in the S phase [63]. Another study showed the arrest of the G2-M phase of the cell cycle in MDA-MB-231 and MCF-7 breast CCL when exposed to 4-Methyl-3-nitro-benzoic acid [64,65]. The efficacy of phenolics against cancer cell proliferation, migration, and invasion is well documented in many literatures [66,67]. *P*-coumaric acid decreased the viability of HCT15 and HT29 colorectal cancer cell lines [68,69]. Caffeic acid and its derivatives are also found to reduce the cell viability of cancer cell lines; caffeic and 5-caffeoylquinic acids reduced the cell proliferation of colorectal (HT-29) and fibrosarcoma cell lines (HT-1080) by modulating the cell cycles at various stages [70,71,72]. The phenol, di-caffeoylquinic acid, also reduced the proliferation of human colon CCC (DLD-1) [73]. Ferulic acid showed inhibition of pancreatic CCL MIA-Pa-Ca-2, and gallic acid inhibited CCL HeLa and HTB-35 [74,75]. Cinnamic acid derivatives with phenyl groups showed cytotoxicity in CCCs HT-29 (human colorectal CCL), A-549 (human lung CCL), MDA-MB-231, and HeLa (cervical CCL). The phenyl-substituted acids showed better efficacy in inhibiting cancer cell proliferation. At 0.1 mM concentration, the phenyl substitutes inhibited 84–92% of the cancer cells compared to non-substituted compounds, showing maximum inhibition of up to 63% [76].

The phenolics are studied for their toxicity in normal human cell lines. Compared to synthetic ones, naturally occurring phenolics showed less toxicity even at higher doses [77].The phytochemical protocatechuic acid was tested for safety and toxicity. Protocatechuic acid showed an LD_50_ value of 800 mg per kg by inter-peritoneal and 3.5 g/kg by intravenous routes [78]. A toxicity assessment of gallic acid (GA) in rats was conducted in which the rats were fed a GA-rich diet (up to 5%) for 13 weeks and no symptoms of toxicity were observed [79]. Similarly, *p*-coumaric acid also exhibited low toxicity, with an LD_50_ ~ 2850 mg/kg body weight [80]. In conclusion, phenolics and their derivatives are safe and have anti-proliferative effects on cancer cell lines. The compounds’ toxicity profile may vary depending on the structure, administration route, and dosage.

### 3.1. Curcumin

Curcumin has shown great potency in chemoprevention, isolated from *Curcuma longa*. The potent compound shows chemopreventive effects through ROS scavenging, signaling pathway modulations, apoptosis induction, and tumor microenvironment regulation. Curcumin is a safe and effective chemopreventive agent with low toxicity to normal cells. One of the essential aspects of curcumin is that it is budget-friendly, yet effective. In Asian countries, curcumin is used deliberately, and the plant’s root is used as a coloring and flavoring agent for food. The compound has many other benefits, such as being anti-inflammatory and having potent antioxidants [81]. The phytochemical is abundant in the spice turmeric and has a mixture of many bioactive compounds. The curcumin derivative in turmeric, tetrahydrocurcumin, has been a great attraction for research due to its anti-cancer effects and excellent solubility in water [82].

Curcumin fights cancer by its action on various essential signaling molecules such as CDKs, NF-kB, tumor necrosis factor-alpha (TNF-a), and cyclooxygenase-2 (COX-2) [83,84]. It shows considerable anti-inflammatory and anti-cancer effects in different clinical and preclinical studies. Many in vitro experiments also demonstrated diverse mechanisms by which curcumin inhibits cancer cells. CDK overexpression is associated with cancer, and breast and skin cancer treatment with curcumin decreases cancer progression by inhibiting CDK4 [85]. Curcumin downregulates gene expression in cancer onset and progression, such as VEGF, angiopoietin, MMP-9, and MMP-3 [86].

### 3.2. Resveratrol

Resveratrol is chemically 3, 5, 40-trihydroxy-trans-stilbenes and is abundant in grapes, berries, and many other plants. The compound has anti-ageing properties and has excellent roles in managing many diseases, including cancer, diabetes, neurodegeneration, arthritis, etc. [87]. Resveratrol modulates different signaling pathways in cancer onset, progression, and metastasis. It is also known to induce programmed cell death, reduce inflammatory responses, and aid in lowering the angiogenesis and conversion of a benign tumor into a malignant tumor [88,89]. The side effects of cancer treatment are significant complications in chemotherapy. Resveratrol has a major advantage: it eliminates the toxicity and side effects of cancer therapies and may be used as a combinatorial treatment [90,91,92]. The phytochemical reduces toxic heavy metals such as arsenic in renal cells. Using resveratrol inhibits the oxidative stress induced by arsenic trioxide, and a decline in arsenic concentration is observed in the hepatic cells [93,94]. The phytochemical is also beneficial in the treatment of acetaminophen-induced liver toxicity and cisplatin-induced kidney disorders [95]. External application of the phytochemical inhibits the effects of UV-B radiation on skin edema and reduces the production of hydrogen peroxide in mice. Extended application of resveratrol showed a tumor reduction and delayed the onset of cancer, whereas short-term application led to cytotoxicity against cancer growth [96,97]. Various research claims that resveratrol treatment modulates the signaling molecules associated with oncogenesis and shows inhibitory effects on cancer cells [98,99].

### 3.3. Apigenin

Apigenin is highly abundant in nature in the form of fruits and vegetables. The phytochemical is a flavone derivative and has anti-angiogenic properties. The properties are related to the modulation of signaling pathways associated with cancer induction, apoptosis, and cell cycle arrest [100]. Various research studies have shown the chemopreventive roles of apigenin in in vivo models. Different animal models were studied with variations in dosage, mode, and frequency of administration of the phytochemical. The major pathway modulated by apigenin is the phosphoinositide 3-kinase (PI3K)/Akt signaling pathway [101]. Apigenin reduces Her2/neu protein expression in mouse models of cancer [102]. The phytochemical shows chemopreventive effects by stimulating apoptotic cell death and cell cycle arrest. The natural phytochemical inhibits the progression of prostate cancer by inhibiting the NF-kB pathway [103]. Apigenin administration in the form of a parsley-rich diet improved antioxidant levels [104]. Other biological activities associated with phytochemicals include reduced plasma levels and platelet aggregation [105].

### 3.4. Gingerol

Gingerol, a phenolic compound, is a major bioactive compound present in ginger. According to a published study, mice treated with gingerol (5 mg/kg body weight) demonstrated inhibition of tumor growth and metastasis of breast cancer cells to other parts of the body by inhibiting caspase-3 expression [106]. Gingerol also inhibits metastatic lung cancer, breast cancer proliferation, metastasis, and invasion by suppressing the AKT and p38MAPK pathways [107].

### 3.5. Thymoquinone

Thymoquinone (TQ) is chemically 2-isopropyl-5-methyl-1,4-benzo-quinone and a bioactive constituent in black cumin seed oil. The compound has been extensively studied in in vivo models. When administered to BALB/c mice at 10 mg/kg, there is a decline in tumor size. TQ showed anti-cancer effects by inducing apoptosis and blocking STAT3 phosphorylation in gastric cancer cells; reduced STAT3 showed a reduction in JAK2 and c-Src activity [108]. Preclinical studies showed the potential role of TQ in combinatorial therapy with other chemotherapeutic agents [109]. BALB/c mice with transplanted breast cancer cells (EMT6/P cell line) were studied for inhibition by TQ along with melatonin. and it was found that it leads to decreased tumor size and cell death induction [110].

## 4. Tannins in Cancer Management

Tannins are high-molecular-weight (500–3000 Dalton), heterogeneous, and water-soluble compounds that are abundant in plants and common in food and beverages [91]. They are highly reactive, and owing to this, they form inter- and intra-molecular hydrogen bonds with other macromolecules such as proteins [111,112]. Tannins are classified into two classes: hydrolysable tannins and condensed tannins. Hydrolysable tannins are further classified into two groups. First are the gallotannins, which yield a sugar and gallic acid (GA) upon hydrolysis, and second are the ellagitannins, which yield an additional ellagic acid when hydrolyzed. The second class of tannins is the condensed tannins, the proanthocyanidins. The proanthocyanidins are highly abundant plant-derived polyphenols [112]. These compounds, unlike the hydrolysable tannins, do not hydrolyze in the presence of weak acid. However, under acidic and alcoholic conditions, they decompose and produce red pigments named phlobaphenes. The high structural complexity and the polymeric nature are responsible for less attention being paid to the tannins [113]. Proanthocyanidins and their monomers have drawn recent attention as they have various human health benefits, namely, antioxidant, anti-cancer, anti-inflammatory, anti-diabetic, etc. [114]. Table 2 lists the tannins and their roles against cancer proliferation.

### 4.1. Epigallocatechin Gallate

Green tea is a rich source of antioxidants and a proven preventive compound for numerous diseases. The major bioactive compound present in green tea is epigallocatechin gallate (EGCG), made up of three bound heterocyclic rings; delocalization of electrons leads to the scavenging of free electrons [115]. The tea catechins that contain the bioactive compound show redox properties with ROS. EGCG also acts as a metal chelating agent and prevents the production of ROS [116,117]. Although the compound is rich in health benefits, it has very low bioavailability, is indigestive, and has efflux properties [118,119,120]. Due to these reasons, EGCG shows a reduced effect in clinical trials. The major signaling pathways modulated by the compound are JAK/STAT, Janus kinases (JAK), signal transducer and activator of transcription proteins (STAT), NF-κB, MAPK, etc. [121,122]. The compounds have proven to exhibit tumor suppression and include genes such as p53, p21, p16, and Rb [123,124].

### 4.2. Gallic Acid

Tannin, or gallic acid (GA), shows anti-proliferative effects on multiple cancers such as lung, prostate, breast, colon, and esophageal cancer [125,126,127] by inducing cell death by apoptosis and other mechanisms. GA has shown antiproliferative effects on various human prostate cancer cell lines, such as LNCaP, PC-3, etc., by modulating multiple mechanisms [128,129]. In an in vivo study on BALB/C male nude mice, xenografts for DU145 and 22Rv1 were administered with GA in water for 6 weeks, and this resulted in reduced tumor size in the mouse models [114]. GA minimizes the proliferation of cancer cells by inducing apoptosis in H446, Calu-6, A549, etc., cell lines [130]. GA also stimulates mitogen-activated protein kinase (MAPK) inhibition, leading to apoptosis induction in lung cancer cells; GA reduced the number of viable NCI-H460 cells through induction of apoptosis and ultimately leading to G2/M phase arrest [131]. In another study, C57 black mice transplanted with LL-2 cells were administered GA (1 mg/mL) ad libitum, and it resulted in a reduction of tumor growth compared with the controls [132]. Nude NCI-H460 xenograft mice were administered GA orally, and it showed a reduction in tumor growth and induced caspases 3, 8, and 9 in the mouse model that induced apoptosis via the caspase-mediated mitochondrial pathway [133]. GA also induces apoptosis in human osteosarcoma cells by modulating the MAPK pathways. The compound shows inhibitory effects on the cancer cell lines U-2OS and HOS osteosarcoma cell lines. GA administration also inhibited the tumor growth in xenografts in a dose-dependent manner by downregulating PCNA and CD31 levels and thereby inducing apoptosis in the tumor cell lines [134].

**Table 2:** Tannins and their roles against cancer proliferation.

Tannins	Cancer	Effect on Cancer	Refs.
Tannic acid (TA)	Breast cancer cell lines (MCF-7, MDA-MB-231, BT474) Prostate Cancer Cells (PC-3 and LNCaP) Head and Neck Cancer (FaDu and YD-38)	Growing cells remodelled collagen caspase-mediated apoptosis MCF7 cells showed sensitivity to the pro-apoptotic effect of TA. TA induced apoptosis in HER-2 positive cell line BT474 Inhibits migration, invasion and ability to form colonies. Expression modulation of cytochromes CYP17A1, 3A4, 2B6, NQO1, GSTM1, and GSTP1. FaDu cells showed cell cycle arrest in G2/M phase. Apoptosis induction with increase of cell population at sub-G1 phase. Both intrinsic and extrinsic cell death was triggered and phosphorylation of kinases of ERK, AKT and PKB	[135,136,137] [138] [139]
Ellagic acid (EA)	Human Bladder Cancer Cell Lines (T24, UM-UC-3, 5637, and HT-1376) Lung Cancer cell line A549	EA exhibits in vitro and in vivo anti-tumor activity for human bladder cancer. Inhibits tumor cell proliferation; migration and invasion. Down-regulation of PD-L1 and reduction of angiogenesis. Inhibition of kinase-related pathways such as PI3K/AKT, PDK3, and SPHK.	[140] [141,142,143]
EGCG	Breast cancer cell line 4T1 Human esophageal squamous carcinoma cells Eca109 Colorectal cancer (DLD-1 and SW480) Oral squamous cell carcinoma (HSC-3)	EGCG induced breast cancer apoptotic cell death at 24 h Caspase 3, 8 and 9 activation. Apoptosis induction by reduced protein expression of adenosine triphosphate binding cassette subfamily G member 2 (ABCG2) and reduction of Bcl-2. Decrease in Wnt-β catenin pathway. Increase in Caspase 3 and 7 activities.	[144] [145] [146] [147]
Gallic acid	Prostate cancer cell lines (DU145) Human lung cancer cells. Calu-6 and A549 Leukemia K562 cell line	Toxicity towards prostate cancer cells compared with DU145 cells. Exhibits apoptotic effects in DU145 cells by stimulating a pre-existing apoptotic pathway. Activates mitogen-activated protein kinase (MAPK) inhibition. BCR/ABL kinase inhibition. NF-Κβ inactivation. Cyclooxygenase-2 (COX-2) Down-regulation.	[127] [130] [148]
Procyanidins	Human breast cancer cell line MCF7 Non-small cell lung cancer (NSCLC)	MCF7 cell proliferation inhibition was observed in a concentration/time-dependent manner. Induced cell cycle arrest and apoptosis. NSCLC cell proliferation inhibition was observed Induced cell cycle arrest and apoptosis	[149] [150]
Green tea catechins	Human lung cancer cell line PC-9 Human prostate cancer DU145 cell line	Inhibited the proliferation of catechins in the order EGCG > ECG (Epicatechin gallate) > EGC (Epigallocatechin)≫EC (epicatechin) Growth reduction of prostate cancer cells DU145 Induction of apoptosis, ROS formation in the order ECG > EGCG > EGC > EC	[151] [152]
Epicatechin (flavon-3-ol monomer units)	Human bladder cancer TCCSUP cell line	20% growth inhibition at 20 µg/mL of EC was observed	[153]

## 5. Alkaloids in Cancer Treatment

Alkaloids are the phytochemicals that possess the most promising anti-cancer activities. The phytochemical class has diverse compounds derived from plants, animals, microbes, and many more [20]. The low molecular weight alkaloids are organic nitrogenous compounds. The compounds in this group are generally colorless and non-volatile and exhibit a low toxic effect on human cells. The action of alkaloids for cancer cell inhibition is to block the action of the topoisomerase enzyme, which further stalls DNA replication and promotes cell death [22]. For these reasons, alkaloids have been used as a parent molecule for designing and developing compounds possessing human health benefits [22]. Various alkaloids having anti-cancer effects include colchicine, vincristine, vinblastine, morphine, etc.

Colchicine is an anti-mitotic agent that prevents microtubule elongation by binding to tubulin and forming a tubulin-colchicine complex reversibly. However, at higher doses, the alkaloid causes significant damage to the normal tissues and limits its use in chemotherapy [96]. Vinblastine sulfate, USP, is obtained from the flowers of a common medicinal plant (*Catharanthus roseus* spp.). The compound shows its anti-cancer effect by halting cell growth at the metaphase [154]. The alkaloid vincristine is also used as an anti-cancer agent. The drug is administered intravenously due to its low bioavailability [150]. Vindesine, marketed as vindesine sulfate, gained FDA approval in 1994. Like other Vinca alkaloids, vindesine blocks the cells in metaphase during mitosis [154]. In vitro studies show that vindesine sulfate inhibits the malignancy and invasion of cancer cells. Vindesine sulfate has more potency than other alkaloid drugs. Vinorelbine is also a semi-synthetic vinca alkaloid sold under the brand name Navelbine [155]. It is a chemotherapeutic drug for treating non-small cell lung cancer that has spread metastatically (NSCLC) [155]. Table 3 lists alkaloids with their pharmacological mechanisms.

## 6. Terpenes in Cancer Treatment

Terpenes are highly abundant phytochemicals and are numerous. The terpenes are found in various sources, such as plants, flowers, and insects. The compounds are responsible for the taste and fragrance of the plants. We can classify terpenes based on the number of isoprene units and their organization [165]. Myrcene, a monoterpene, and the sesquiterpenes β-caryophyllene and α-humulene are the terpenes most common. Myrcene extracts have shown cytotoxic effects in cancer cell lines such as breast and colon cancer [166]. The terpene β-cp exhibits cytotoxic potential against lung and ovarian cancer cell lines by inducing cell cycle arrest and apoptosis [167,168]. The compound shows anti-proliferative effects in a glioblastoma model. β-cp at 20 µM induces proapoptotic and antiproliferative effects by modulating the JAK/STAT pathway in osteosarcoma cells [169]. Glycyrrhizin (Gy), a triterpene glycoside, is the active constituent found in the licorice root of *Glycyrrhiza glabra*. BALB/c nude mice xenografts of A549 cells (lung cancer) were transfected with TxA2 receptor (TPa), Gy with a dosage of 135 mg/kg, which reduced thromboxane synthase and PCNA expression by suppressing the TxA2 pathway [170]. It has anti-cancer and antioxidant activities. Gy also enhances NO production by stimulating with interferon-gamma (IFN-γ), and high NO concentrations are associated with cancer cell death [171]. Table 4 shows some of the terpenes and the anti-cancer potential of the compounds.

## 7. Mechanism of Action of Phytochemicals

**Figure 4:**
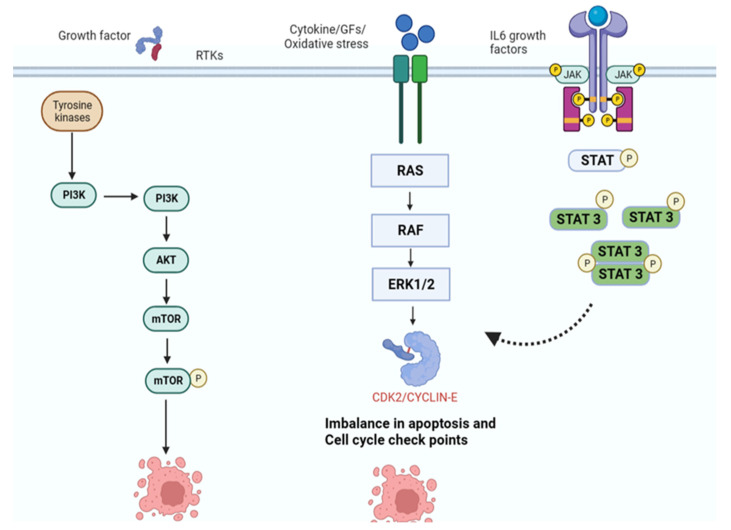
Different signaling pathways targeted by the phytochemicals responsible for the onset of cancer and cancer proliferation, metastasis, and invasion.

The phytochemicals exert deleterious effects on cancer cells through various mechanisms, including modulations in signaling pathways and the onset of apoptosis [206]. The anti-cancer agents show their effects by blocking the generation of carcinogenic species and obstructing the interaction between carcinogens and cells, thus delaying tumor formation [207]. The signaling pathways majorly associated with cancer are the mitogen-activated protein kinase (MAPK) pathway, nuclear factor kappa B (NF-Kb), and activator of transcription proteins (STAT) pathway. The signaling pathways are modulated so that they may be overactivated or blocked and govern the metabolic pathways in various cancers. The modulations further lead to cancer onset and proliferation, and they promote various hallmarks of cancer such as angiogenesis, increased glycolysis, and metastasis [9]. The signaling pathways and modulated enzymes and factors are a major target to inhibit or activate for the therapeutic treatment of cancers [23]. The MAPK pathway is associated with the onset of tumors such as melanomas and is a target for inhibiting the treatment of related cancers. Various phytochemicals such as quercetin, curcumin, ellagic acid, rosmarinic acid, etc. [208]. Refs. [209,210,211] are associated with halting the MAPK pathway, shown in Figure 4. Quercitin has demonstrated inhibitory effects on human hepatoma cell lines HepG2 by blocking the ERK pathway and phosphatidylinositol-3-kinase (PI3K)/Aurora kinase B (AKB) pathways [212]. Gallic acid showed a deleterious effect on the invasiveness of mouse brain endothelial cells and glioblastoma cells, U87 and U251, by blocking some pathways involved in cancer progression [213]. NF-kB pathway has major roles in cancer development and progression, promoting the proliferation of cancer cells, aiding metastasis, and skipping apoptosis. The phytochemicals have shown inhibitory effects against NF-kB; the phytochemicals involved are capsaicin, ursolic acid, gingerol, eugenol, etc. [214].

Apoptosis is an essential process of programmed cell death and significantly eliminates tumor cells [22,215]. Phytochemicals have been shown to induce apoptotic effects on cells by upregulating caspase 3 and 9 expressions, decreasing the growth and development of colorectal cancer and lung cancer [216]. The phytochemicals involved in apoptosis are punicalagin and 5-methoxyangenylalkanni. Apigenin, a flavonoid derivative, is associated with the modulation of the kinase pathway and blocks the cells in the G2/M phase. Apigenin can inhibit the growth of HepG2 cells [217]. Esculetin induces apoptosis in various human cancer cell lines, including HSC4, HSC4 oral squamous cell carcinoma, the leukemia cell line U937, and melanoma cells G361 [218]. The phytochemical is also a potent inhibitor of the Wnt-β-catenin pathway. It blocks the formation of the β-catenin-Tcf complex, suppressing colon cancer cell proliferation [219]. In colon cancer, the phytochemical diosgenin s apoptosis by increasing caspase 3 activity, inhibiting Bcl-2 [220]. The phytochemical artabotryside A induces apoptosis in U87 cells by arresting the cell cycle at the G2/M phase of the cell cycle [221]. Caffeic acid-induced apoptosis was induced in the breast cancer cell T47D by activation of the Fas/FasL pathway [222]. Several other phytochemicals are known to induce anti-cancer effects by inducing apoptosis, such as lutein, capsaicin, rhein, etc.

Cell cycle progressions are associated with activating cyclin-dependent kinases (CDKs). The levels of CDKs are regulated by cyclin-dependent kinase inhibitors (CKIs), which maintain the level of CDKs [223]. Various phytochemicals, such as mangiferin [224], naringenin [225], berberine [226], fisetin [227], etc., have demonstrated inhibitory potentials for the progression of the cell cycle. Ferulic acid from *Allium cepa* has been studied to elevate the expression of genes associated with the association of centrosomes and arrest the cell cycle at the synthesis (S) phase, which results in the inhibition of colon cancer Caco-2 cells [228]. Withaferin A, isolated from *Withaniasomnifera* spp., arrests the cell cycle at the G2/M phase by lowering CDK levels in various cancer cell lines [229]. In addition, other phytochemicals such as capsaicin, kaempferol, and berberine induce arrest in the cell cycle. Cancer-related epigenetic variations are associated with chemical changes to histones and gene expression. DNA’s hyper- and hypomethylation leads to chromatin condensation and tumor inhibitory gene inhibition. Improper oncogene expression is also the result of methylated cytosines [230].

## 8. Conclusions and Future Perspectives

Phytochemicals have emerged as a major source for developing novel leads for drug discovery and development. An advanced approach to combining traditional knowledge with the drug discovery process can lead to the discovery of novel compounds that can aid in the management of various life-threatening diseases. The advancements in analytics and bioinformatics have also facilitated the entry of new leads from plants into the evaluation process. Cancer is a complex and hard-to-treat disease with various complications. The conventional methods of cancer therapeutics have a lot of drawbacks, such as side effects, chemoresistance, and reversal of cancer. The utmost need is to develop more potent therapeutic compounds with the least toxicity. Using phytochemicals in combination with current methods of cancer treatment can aid in reducing the effects of cancer. The phytochemicals work on cancer cells by modulating the cell signaling mechanism and inducing apoptosis in the cancer cells. Various phytochemicals have shown their anti-cancer effects in in vivo, in vitro, and clinical trials.

Detailed studies at preclinical and epidemiological levels are needed to identify more such beneficial compounds and their use against cancer alone as well as in combination with other drugs already available.

## Figures and Tables

**Table 1:** Some of the phytochemicals used against cancer, the methodology, and the final outcomes.

Phytochemical	Cancer	Interventions	Effect	References
*Allium sativum*	Colorectal, liver, and pancreatic cancer patients Colorectal ademas	500 mg of aged garlic extract (GE) in 4 capsules for 12 weeks 2.4 mL GE in 3 capsules twice a day for 1 year	Natural killer (NK) cells increased in number and activity. Reduced size and number of colon adenomas.	[40] [41]
Camptothecin (Ct)	Patients with refractory cancer Primary/metastatic lung cancer patients	Ct: 3 weeks drug-1-week rest; Nitro-Ct: 5 day drug- 2 days rest 6.7–26.6 µg/kg of Ct in the form of aerosolized liposomes were given 5 days a week for 6 weeks, followed by a gap of 2 weeks.	Both the compounds showed tumor regression in patients with breast cancer, prostate cancer, and melanomas. 3 lung patients stabilized upon dosage.	[42] [43]
Curcumin	Urinary bladder cancer, uterine cervical neoplasm, and intestinal metaplasia Advanced pancreatic cancer	500 mg/day, orally, for 3 months Dosage was 8 g/day for one month	Improvement in 1 out of every 2 patients with bladder cancer and 1 out of 6 patients with intestinal Metaplasia, and 1 out of 4 patients with uterine cervical neoplasm. Study was conducted on 21 patients, of whom 1 had stable disease for >18 months and 1 had tumor reversion.	[44] [45]
Green tea	Patients with high-grade prostate intraepithelial neoplasia Patients with adenocarcinoma of the prostate Esophageal cancer Patients with colon, rectum and pancreas cancer	Green tea catechins (600 mg) were given daily, orally, for one year Tea consumption as a daily routine Usual green tea consumption Non-regular tea consumption	Improved quality of life Risk declination of prostate cancer with increased consumption of green tea. Reduced risk of Esophageal cancer. Inverse relation was associated with cancer and green tea consumption.	[46] [47] [48] [49]
*Panax ginseng*	Patients with cancer of uterine, ovary, rectum, stomach, etc	3000 mg/day of the heat-processed ginseng for 12 weeks	Improvement of mental and physical functioning, and hence improved quality of life.	[50]
Isoflavones	Prostate cancer	(60 mg) daily for 12 months	Reducing prostate cancer incidence for patients aged 65 or more.	[51]
Synthetic genistein	Prostate cancer	54 patients with localized prostate cancer. (30 mg) daily for 3–6 weeks	Decreasing level of serum prostate specific antigen (PSA).	[52]
Soy isoflavone	Prostate cancer	86 patients with localized prostate cancer. (80 mg total isoflavones, 51 mg aglucon units) daily for 6 weeks	No significant change in serum hormone levels, total cholesterol, or PSA.	[53]
Flavonoid mixture	Colorectal cancer	(20 mg apigenin and 20 mg EGCG) for 3–4 years. 87 patients with resected colorectal cancer or polypectomy	Reducing the recurrence rate of colon neoplasia in patients with resected colon cancer.	[54]
Isoflavones and curcumin	Prostate cancer	Isoflavones (40 mg) and curcumin (100 mg) daily for 6 months	decreasing level of serum PSA.	[55]

**Table 3:** Alkaloids and their therapeutic effect and pharmacological mechanism.

Alkaloid	Pharmacological Mechanism	Therapeutic Effect	Refs.
Vinblastine	-Binds to tubulin and prevents microtubules from binding. -Induce apoptosis and mitotic death.	Cervical cancer Breast cancer Lung cancer Head and neck cancer Hodgkin’s lymphoma Testicular cancer	[156,157]
Vincristine	-Binds tubulin dimer. -Prevents microtubule structure formation.	Acute myeloid leukemia (AML, ANLL) Acute_lymphoblastic leukemia (ALL) Hodgkin’s_lymphoma Non-Hodgkin’s lymphoma	[158,159]
Vindesine	Possess anti-mitotic activity	Melanoma Lung cancers Uterine malignancies	[154]
Vinorelbine	Exhibits broad-spectrum antitumor activity. Antineoplastic activity	Breast cancer Non-small cell lung cancer (NSCLC)	[159,160]
Vinflunine	Decreases metaphase to anaphase transition, Prevents cancer cells from entering mitosis. Increases apoptosis	Metastatic Urothelial carcinoma Transitional cell carcinoma Breast cancer	[161]
Colchicine	Microtubule destabilizers perturb the assembly dynamics of microtubules.	Gastric cancer	[162,163]
Colcemid	Mitotic arrest Kinase inhibition	Lung Cancer	[164]

**Table 4:** Anti-proliferative role of terpenes in cancer.

Terpene	In Vitro Effects	In Vivo Effects	Clinical Trials	Refs.
Myrcene	Cytotoxic effects on cancer cell lines Reduced DNA damage	Carcinogenic at higher doses	N/A	[172,173,174,175]
Limonene	Shown cytotoxic effects Mediates cell cycle arrest Decreased migration and invasion of cancer cells Apoptosis and autophagy induction Inhibition of the PI3K/Akt pathway	Decreased tumor growth and metastasis, c-jun, and c-myc expression Induced apoptosis and latency period.	Decreased the expression of proteins involved in tumor progression.	[176,177,178,179,180,181,182,183]
Pinene	Reduced cell viability. Induced apoptosis, ROS production, and cell cycle arrest	Reduced the number and growth of tumors.	N/A	[184,185,186,187]
Elemene	Induced cell cycle arrest and apoptosis Inhibited MAPK pathway Reduced tumor migration and invasion Inhibited angiogenesis	N/A	Effective agents in chemotherapy. Reduced toxicity of chemotherapy.	[188,189,190,191,192,193,194]
Terpinene isomers	Reduced proliferation and induced apoptosis in cancer cells	N/A	N/A	[195,196,197,198]
Valencene	Reduced cellular proliferation and acted efficiently synergistically with doxorubicin	N/A	N/A	[199,200]
Nerolidol	Exhibited cytotoxic effects and induced apoptosis and cell cycle arrest. Acted synergistically with doxorubicin	Inhibited cancer growth	N/A	[201,202,203,204,205]

## Data Availability

All the associated Data is contained within the article.
